# Probiotic Roles of *Clostridium butyricum* in Piglets: Considering Aspects of Intestinal Barrier Function

**DOI:** 10.3390/ani14071069

**Published:** 2024-03-31

**Authors:** Xiaopeng Tang

**Affiliations:** State Engineering Technology Institute for Karst Desertification Control, School of Karst Science, Guizhou Normal University, Guiyang 550025, China; tangxiaopeng110@126.com; Tel.: +86-155-0850-9218

**Keywords:** *Clostridium butyricum*, intestinal barrier function, intestinal microorganisms, intestinal immunity, weaned piglets

## Abstract

**Simple Summary:**

*Clostridium butyricum* (*C. butyricum*) is a Gram-positive obligate anaerobic bacillus with strong heat resistance, acid resistance, and bile-salt tolerance, which lays a foundation for its application in the feed industry. Previous studies have demonstrated that *C. butyricum* plays a significant role in regulating the intestinal health of weaned piglets. In general, *C. butyricum* promotes intestinal health by regulating the functions of the mechanical barrier, chemical barrier, immune barrier, and microbial barrier of piglets.

**Abstract:**

China, as the global leader in pork production and consumption, is faced with challenges in ensuring sustainable and wholesome growth of the pig industry while also guaranteeing meat food safety amidst the ban on antibiotics usage in animal feed. The focus of the pig industry lies in guaranteeing piglet health and enhancing overall production performance through nutrition regulation. *Clostridium butyricum* (*C. butyricum*), a new type of probiotic, possesses characteristics such as heat resistance, acid resistance, and bile-salt tolerance, meaning it has potential as a feed additive. Previous studies have demonstrated that *C. butyricum* has a probiotic effect on piglets and can serve as a substitute for antibiotics. The objective of this study was to review the probiotic role of *C. butyricum* in the production of piglets, specifically focusing on intestinal barrier function. Through this review, we explored the probiotic effects of *C. butyricum* on piglets from the perspective of intestinal health. That is, *C. butyricum* promotes intestinal health by regulating the functions of the mechanical barrier, chemical barrier, immune barrier, and microbial barrier of piglets, thereby improving the growth of piglets. This review can provide a reference for the rational utilization and application of *C. butyricum* in swine production.

## 1. Introduction

The animal intestinal tract acts as a protective barrier, allowing for the absorption of nutrients while safeguarding the body against harmful chemicals from both internal and external sources [1,2,3]. The integrity of the intestinal barrier is essential for the digestion and absorption of nutrients, playing a vital role in maintaining animal health. However, in swine production, various factors such as weaning stress [4], heat stress [5], pathogen infection [6], mycotoxin [7], lipopolysaccharide [8], and diquat [9] can cause damage to the intestinal mucosa and disrupt intestinal mucosal homeostasis, negatively impacting animal growth and development. Traditionally, antibiotics have been used in animal feed as growth and health promoters, but they have had serious detrimental effects on human health and environmental safety [10,11]. As a result, many countries, including China, have prohibited the use of antibiotics in animal feed. Therefore, finding antibiotic alternatives that are safe and pose no potential threats has become a major concern in the field of animal nutrition [12,13,14,15].

Probiotics are living bacteria with physiological activity. Numerous in vivo and in vitro studies have confirmed that probiotics can improve the balance of microbes in the intestinal environment, enhance immune function, and benefit intestinal morphology [16,17,18,19,20,21,22]. In all of these studies, *Clostridium butyricum* (*C. butyricum*) has been identified as an effective probiotic that promotes animal growth and maintains intestinal barrier function [20,23,24,25,26,27,28,29]. *C. butyricum*, also known as *Clostridium tyrosine*, is a Gram-positive obligate anaerobic bacillus first isolated from pig intestines in 1880 by Prazmowski. It is a common gut commensal bacterium in humans and animals and can be found in soils and healthy intestines [30,31]. *C. butyricum* can produce butyric acid, which plays a crucial role in energy metabolism and the development of normal intestinal epithelial cells [32]. Due to its resistance to low pH, high temperature, and high bile-salt concentrations, *C. butyricum* has potential as a feed additive [33,34]. The use of *C. butyricum* as probiotics in swine has been gaining attention for its ability to produce short-chain fatty acids (SCFAs), amino acids, enzymes, and vitamins [35,36], which can improve the growth performance, feed efficiency, antioxidant capability, immune function, and intestinal microflora balance of pigs [20,26,27,28,37]. While studies on the effects of *C. butyricum* on the growth and gut health of piglets are scattered, few have aggregated these findings into a single review. This study aims to review the probiotic role of *C. butyricum* in piglet production, specifically focusing on intestinal barrier function, to provide guidance on the proper utilization and application of *C. butyricum* in swine production.

## 2. *Clostridium butyricum* and Intestinal Physical Barrier

### 2.1. Clostridium butyricum Promotes Intestinal Development

The exchange of gases and nutrients between the body and the external environment is crucially facilitated by the intestinal tract, which additionally assumes the task of digesting and absorbing nutrients while functioning as a selective barrier to prevent harmful substances from entering the body [2,38]. In practical production, piglets often face numerous stressors, including the challenges of weaning and the detrimental effects of oxidative reactions, which can result in structural damage to the intestinal mucosa and impair intestinal barrier function [4,39]. As a result, piglets may exhibit decreased feed consumption, reduced daily weight gain, and an increased risk of diarrhea, even leading to mortality in serious cases [40,41]. Therefore, it is imperative to uphold the well-being of the intestinal tract to ensure optimal health and productivity for these animals.

Weaning is a crucial stage for piglets but can also cause weaning stress because of dietary changes, environmental adjustments, and other factors. Weaning stress can lead to intestinal mucosa atrophy, cell apoptosis, and significant impacts on the intestinal morphology of piglets [42,43,44]. Measurements of crypt depth (CD), villus height (VH), and the VH-to-CD ratio (VCR) are important indicators of intestinal growth and function [45,46]. Previous studies have shown that the dietary inclusion of *C. butyricum* can enhance intestinal morphology and structure, improve intestinal development, and subsequently improve the intestinal absorption and digestion functions of piglets [47,48,49]. For example, Wang et al. [50] observed that piglets fed with 6 × 10^9^ CFU/kg *C. butyricum* had a significantly increased jejunal VCR compared to control piglets when challenged with lipopolysaccharide (LPS). Wang et al. [51] found that piglets fed with *C. butyricum* (6 × 10^9^ CFU/kg) had a significantly increased jejunal VH and VCR and a decreased jejunal CD compared with control piglets. Li et al. [33] showed that diets supplemented with 5 × 10^5^ CFU/g *C. butyricum* significantly reduced intestinal CD and increased the VCR of piglets challenged by enterotoxigenic *Escherichia coli* (ETEC) K88, indicating that *C. butyricum* was beneficial to intestinal health. Furthermore, Wu et al. [37] confirmed that feeding piglets a diet with 1.44 × 10^9^ CFU/kg *C. butyricum* SLZX19-05 resulted in a significant increase in the VH and VCR, as well as a decrease in CD in the jejunum and ileum of piglets.

In conclusion, research has demonstrated that *C. butyricum* has a positive impact on the intestinal development of piglets by enhancing intestinal morphology and structure. This suggests that the inclusion of *C. butyricum* in the diet of piglets may be beneficial for their overall health and productivity.

### 2.2. Clostridium butyricum Reduces Intestinal Permeability

The intestinal tract is an essential organ that responds to external stimulation. Stressful conditions, such as weaning stress and infections, can cause intestinal mucosal atrophy, deeper crypts, heightened apoptosis of intestinal mucosal epithelial cells, and increased intestinal permeability [43,52,53]. Intestinal permeability is an important indicator that reflects the intestinal integrity of animals. Increased intestinal permeability allows antigenic compounds to pass the intestinal mucosal barrier, enabling pathogenic bacteria and poisons to translocate and weaken the intestinal barrier function [43,54]. Endotoxins, diamine oxidase (DAO), and D-lactic acid levels in the blood are commonly used to assess intestinal permeability, which could directly indicate the degree of intestinal epithelial mucosa damage [48]. Therefore, higher levels of endotoxin, D-lactic acid, and DAO in the blood indicate increased intestinal permeability. *C. butyricum* has a good regulatory effect on intestinal permeability. For example, Pang et al. [55] indicated that serum endotoxin and D-lactic acid content significantly reduced when piglets were fed with *C. butyricum* (500 mg/kg), and the effect was comparable to a pharmacological dose of zinc oxide (3000 mg/kg). Li et al. [28] showed that dietary supplementation with *C. butyricum* (5 × 10^5^ CFU/g) reduced serum DAO and D-lactic acid levels in ETEC K88-infected pigs. Lu et al. [56] showed that the serum D-lactic acid level decreased when piglets were fed a diet containing 500 mg/kg *C. butyricum*. Fu et al. [48] demonstrated that dietary supplementation with *C. butyricum* (1 × 10^8^ CFU/kg) significantly reduced serum DAO and D-lactate levels in piglets compared to piglets fed a basic diet, indicating that intestinal integrity was improved. These studies revealed that *C. butyricum* supplementation can dramatically reduce intestinal permeability.

### 2.3. Clostridium butyricum Promotes Intestinal Tight Junctions

Tight junctions (TJs) are multiprotein complexes located on the apically lateral membranes of intestinal epithelial cells, primarily composed of Occludin, Claudins, Zonula Occludens (ZO-1, ZO-2, and ZO-3), Myosin light chain kinase (MLCK), actin (F-actin), and Myosin. These proteins play crucial roles in protecting the intestinal physical barrier [2,43,57]. The functionality of the intestinal physical barrier can be indicated by the expression levels of intestinal TJ proteins such as ZO-1, Claudin-1, and Occludin. Previous studies have shown that *C. butyricum* has the ability to enhance the expression of intestinal TJ proteins in piglets, thereby preserving the integrity of the physical barrier and ensuring its normal functions [28,48,56]. For example, Li et al. [28] discovered that the addition of *C. butyricum* to the diet resulted in an increase in the expression of intestinal TJ proteins (ZO-1, Claudin-3 and Occludin) in ETEC K88-infected pigs. Similarly, Lu et al. [56] found that *C. butyricum* had a significant effect on upregulating the expression of genes associated with intestinal TJ proteins (*ZO-1* and *Occludin*) in piglets. Furthermore, Fu et al. [48] observed that piglets supplemented with *C. butyricum* ZJU-F1 exhibited a notable increase in intestinal TJ proteins (ZO-1, Claudin-1, and Occludin) in the jejunum and ileum of piglets. Additionally, Wu et al. [37] confirmed that supplementing with *C. butyricum* significantly increased the expression of *Claudin-1*, *Claudin-2*, *Claudin-3*, and *ZO-1* genes and Claudin-3 protein in the colonic mucosa of piglets.

To summarize, *C. butyricum* has demonstrated its ability to positively regulate intestinal physical barrier function in piglets (Table 1). *C. butyricum* regulates the intestinal physical barrier in the following ways: (i) it enhances VH and the VCR and decreases CD in piglets, thereby maintaining intestinal morphology; (ii) it significantly reduces intestinal permeability and effectively inhibits the intrusion of harmful bacteria; and (iii) it promotes the expression of TJ proteins to uphold the integrity of the physical barrier.

## 3. *Clostridium butyricum* and Intestinal Chemical Barrier

The intestinal mucus layer consists mainly of mucins (MUCs), antimicrobial proteins, digestive enzymes, and microbial metabolites (such as SCFAs), which separate the microorganisms in the intestinal cavity from the epithelial cells, effectively preventing toxins from penetrating the intestine and preventing the invasion of pathogenic bacteria [43,61,62]. Previous studies have shown that *C. butyricum* has a positive effect on intestinal chemical barrier function in many animals, such as pigs [37,48], rabbits [63], broilers [64,65], and mice [66].

The secretion of intestinal MUCs and the activity of intestinal digestive enzymes in piglets decreases during weaning, resulting in a weakened chemical barrier function and an enhancement of intestinal susceptibility [67,68]. *C. butyricum* can effectively regulate intestinal chemical barrier function through the following mechanisms:

(i) *C. butyricum* can stimulate the expression of MUC genes in the intestinal tract of piglets and enhance the secretion of intestinal MUCs. For instance, Fu et al. [48] showed that piglets fed a diet containing *C. butyricum* showed a significant increase in the gene expression of intestinal MUCs (*MUC1*, *MUC4*, and *MUC20*).

(ii) *C. butyricum* can enhance the intestinal chemical barrier by increasing the endogenous digestive enzyme activity of piglets. For example, Hu et al. [27] isolated a strain of *C. butyricum* LY33 from the intestinal contents of healthy pigs and fed it to weaned piglets. They showed that *C. butyricum* LY33 effectively enhanced the activities of duodenal amylase and protease, as well as jejunal amylase, lipase, and protease activities in pigs. Similar results were observed by Lu et al. [56] and Fu et al. [48], who found that piglets fed with a diet containing *C. butyricum* showed significantly increased intestinal amylase, lipase, and protease activities compared to those fed with a basal diet.

(iii) *C. butyricum* has the capacity to inhibit pathogenic bacteria proliferation and preserve intestinal mucosal homeostasis in pigs by boosting antimicrobial peptide (AMP) gene expression. AMPs are a kind of innate immune effector with diverse structures, broad-spectrum and efficient antibacterial activity, and multiple biological functions, such as antibiofilm, immune-regulatory, and anti-inflammatory activity [69,70]. Fu et al. [48] demonstrated that the dietary supplementation of *C. butyricum* ZJU-F1 significantly increased the mRNA expression of AMPs such as *pBD1*, *pBD2*, *pBD3*, and *PR-39* in the jejunum of piglets, and Wu et al. [37] confirmed that the dietary supplementation of *C. butyricum* significantly increased *PR39* gene expression in the colon of piglets.

(iv) *C. butyricum* can enhance the amount of SCFAs in the intestinal tract of piglets, hence maintaining intestinal mucosal homeostasis. SCFAs are metabolites of intestinal microorganisms that provide energy to intestinal epithelial cells and play an important role in epithelial cell integrity, immunity regulation, and pathogenic microorganism inhibition [71,72]. For instance, Zhang et al. [73] demonstrated that 0.1% *C. butyricum* supplementation raised butyrate concentrations and tended to increase propionate and total volatile fatty acids (VFAs) in the feces of weaned piglets. Han et al. [47] discovered that dietary supplementation with 2.5 × 10^8^ CFU/kg *C. butyricum* significantly raised the acetic, propionic, and butyric acid levels and total SCFA concentration in the colon of weaned piglets. López et al. [74] showed that the dietary supplementation of 2.5 × 10^8^ CFU/kg *C. butyricum* significantly increased butyric acid concentration in the feces of weaned piglets.

## 4. *Clostridium butyricum* and Intestinal Immune Barrier

*Clostridium butyricum*, a new bioviable bacterial preparation, can activate the immune system of the host and enhance immune function, thereby maintaining animal health [75,76,77]. In piglets, *C. butyricum* can directly stimulate the intestinal mucosal immune response and improve immune barrier function [48,51]. Firstly, *C. butyricum* can activate the toll-like receptor (TLR)2/TLR4-myeloid differentiation factor 88 (MyD88)-nuclear transcription factor-κB (NF-κB) signaling pathway to stimulate the intestinal mucosal immune response of piglets, hence improving the recognition and transmission ability of pathogens [26,48,51,58]. TLRs are phylogenetically conserved innate immune mediators that can identify gut microbiota and respond to harmful microbes [78,79]. TLR2 and TLR4 are two important members of TLRs, which participate in the immune response mainly by activating the MyD88 pathway to induce the secretion of inflammatory cytokines [51,58,80]. MyD88 is a key adapter protein in the TLR signaling pathway that can activate NF-κB, boosting the production of proinflammatory cytokines and eliciting an immunological response in the intestinal mucosa [51,81]. For instance, Fu et al. [48] showed that *C. butyricum* ZJU-F1 significantly upregulated the gene and protein expression of TLR2, MyD88, and NF-κB in porcine small intestinal epithelial cells (IPEC-J2), as well as the expression of proinflammatory cytokines such as tumor necrosis factor α (TNF-α), interleukin (IL)-1β, IL-6 IL-8, and anti-inflammatory cytokine *IL-10* genes in IPEC-J2 cells and the ileum of weaned piglets. Similarly, Wang et al. [51] demonstrated that dietary *C. butyricum* supplementation dramatically increased the protein expression of TLR4, MyD88, and NF-κB in the jejunal of weaned piglets. On the contrary, Wu et al. [37] showed that dietary *C. butyricum* supplementation significantly reduced the protein expression of p65 NF-κB in the nucleus of ileal mucosa as well as the gene expression of *TNF-α* and *IL-1β* in the ileal mucosa of piglets. Wang et al. [50] showed that dietary *C. butyricum* supplementation substantially reduced TLR4, MyD88, and NF-κB protein expression in the jejunal of weaned piglets challenged with LPS. This is because proinflammatory factors have dual effects: an appropriate amount can regulate the immune response and resist or clear pathogen infection [48]; meanwhile, proinflammatory cytokines can also interact with transforming growth factor -β (TGF-β) secreted by various intestinal cells, jointly promoting the secretion of immunoglobulin A (IgA), IgG, and IgM to maintain intestinal health [58]. However, excessive levels of proinflammatory cytokines can harm intestinal tissue and disturb the body’s immunological balance [48]. Therefore, when piglets are exposed to significant stress, such as LPS stimulation, *C. butyricum* can alleviate intestinal inflammation by inhibiting the TLR4-MyD88-NF-κB pathway, reducing the expression of proinflammatory factors (TNF-α, IL-1β, IL-6, and IL-8) and promoting the secretion of anti-inflammatory factors (IL-10 and TGF-β1) and immunoglobulins (IgA, IgG, and IgM) [26,28,37,49,50].

Secondly, *C. butyricum* can activate cysteine aspartase (caspase1) by increasing the gene expression of the nucleotide-binding oligomerization domain (NOD)-like receptors (NLRs) family pyrin domain, containing 3 (*NLRP3*), *NLRP6*, and *NLRP12,* in the jejunum of piglets to regulate the maturation and secretion of IL-1 family cytokines, and thus reduce intestinal inflammation of piglets [28,60]. NLRPs are a large class of pattern-associated molecular patterns involved in innate immunity, among which NLRP3, NLRP6, and NLRP12 are highly expressed in the small intestine as negative feedback regulators of intestinal inflammation. These proteins play an important role in maintaining the integrity of the mucosal barrier function and promoting symbiosis among gut microorganisms [82,83,84]. Upon recognition of their cognate ligands, NLRPs can assemble into multiprotein complexes known as inflammasomes, which play a pivotal role in activating caspase-1, subsequently leading to the maturation and secretion of IL-1 family cytokines (IL-1β, IL-18, and IL-33) [28,60]. Therefore, on the one hand, *C. butyricum* can enhance intestinal immune response and reduce excessive intestinal inflammation through the bidirectional regulation of the TLR2/TLR4-MyD88-NF-κB signal transduction pathway. On the other hand, *C. butyricum* can stimulate the production of anti-inflammatory cytokines and immunoglobulins and suppress the generation of proinflammatory cytokines, which jointly maintain the intestinal immune barrier of piglets.

## 5. *Clostridium butyricum* and Intestinal Microbial Barrier

Newborn piglets develop a diverse microbiota in their gastrointestinal tract through the consumption of breast milk and exposure to the external environment [85]. The various gut microbiota organisms interact and limit each other, creating a gut microbiota system that acts as the initial line of defense for the gastrointestinal tract. The intestinal microbial barrier plays a pivotal role in preserving the normal physiological activities of the gastrointestinal tract and safeguarding it from potential pathogen attacks [86,87,88]. *C. butyricum* can maintain or restore the dominant intestinal flora of the host, promote the growth and reproduction of beneficial bacteria such as *Lactobacillus* and *Bifidobacterium*, and inhibit the growth of harmful bacteria such as *Salmonella* and *Escherichia coli*, thereby maintaining the intestinal microbial homeostasis of animals [47,48,73,87]. The possible mechanisms through which *C. butyricum* regulates the intestinal microbial barrier of animals include: (i) *C. butyricum* can compete with conditioned pathogens for adhesion sites and nutrients, thereby inhibiting the adhesion and colonization of pathogenic microorganisms within the intestinal tract [89,90,91]; (ii) the polysaccharide decomposition enzyme secreted by *C. butyricum* can decompose polysaccharides into oligosaccharides, thus providing an abundant fermentation substrate for beneficial bacteria, in turn promoting the growth and proliferation of these probiotic microorganisms [92,93]; and (iii) *C. butyricum* can produce a large number of SCFAs, especially butyric acid, which can regulate the intestinal pH value, thus promoting the proliferation of beneficial bacteria while inhibiting the growth of pathogenic bacteria [94].

During the transition period from lactation to weaning, piglets experience significant alterations in their intestinal flora structure due to changes in diet, living environment, and social structure [95,96,97]. *C. butyricum* can improve the richness of intestinal microorganisms and optimize the microecological environment in weaned piglets, promoting a healthier gut microbiota balance [26,48,51]. The effects of *C. butyricum* on intestinal microorganisms of piglets are summarized in Table 2, in which we can see *C. butyricum* plays a crucial role in maintaining the intestinal microecological balance of weaned piglets. It increases the abundance and quantity of beneficial bacteria while inhibiting the colonization of conditioned pathogens. By regulating the structure of the intestinal flora, *C. butyricum* helps to preserve the homeostasis of intestinal microorganisms, promoting a healthy gut environment for piglets.

## 6. Discussion and Prospect of the Application of *Clostridium butyricum* in Piglets

**Figure 1:**
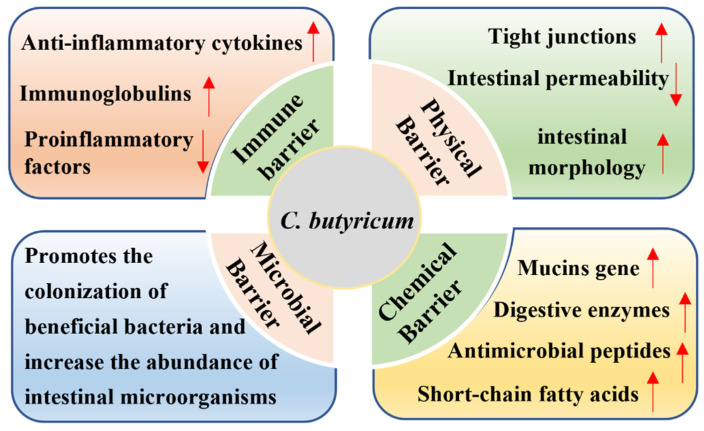
Roles of *C. butyricum* on intestinal barrier function of piglets. *C. butyricum* promotes intestinal barrier function by affecting mechanical barrier, chemical barrier, immune barrier and microbial barrier.

Through the above analysis, we can see that *C. butyricum* has a good regulatory effect on the intestinal tract of weaned piglets. First of all, *C. butyricum* can maintain the good intestinal morphology and proper intestinal permeability of weaned piglets and promote the intestinal physical barrier by promoting intestinal TJ protein expression [26,33,37,48,58,59,60], which provides an important defense line for intestinal resistance to external stimuli. Secondly, *C. butyricum* can promote the secretion of intestinal MUCs, AMPs, digestive enzymes, and SCFAs, thereby improving intestinal chemical barrier function [27,37,47,48,56,73,74], and effectively preventing toxins from penetrating the intestine and preventing the invasion of pathogenic bacteria. Thirdly, *C. butyricum* can enhance intestinal immune response and reduce excessive intestinal inflammation by promoting the production of anti-inflammatory cytokines and immunoglobulins and suppressing the generation of proinflammatory cytokines [28,37,48,49,50,51,60], thereby improving intestinal chemical barrier function, effectively preventing toxins from penetrating the intestine, and preventing the invasion of pathogenic bacteria. Finally, *C. butyricum* can increase the diversity and abundance of intestinal microorganisms, promote the colonization of beneficial bacteria inhibit the colonization of conditioned pathogens in the intestines [26,27,51,89,90,91,92,93,94,95,96,97,98,99,100], so as to improve microbial barrier function and promote a healthy gut environment for piglets (Figure 1).

A healthy gut is key to the growth and development of animals, including piglets. Piglets that experience weaning stress are usually characterized by loss of weight, post-weaning diarrhea due to the immature development of the gastrointestinal tract, and therefore, reduced feeding and nutrient absorption [43]. Many studies have shown that *C. butyricum* can promote the performance of weaned piglets, which is presented in Table 3. 

The improved growth performance observed by dietary *C. butyricum* supplementation might be associated with its promotion of intestinal health. For example, Chen et al. [26] showed that dietary supplementation with 0.4% *C. butyricum* significantly improved the intestinal morphology of piglets, and the feedback on growth performance was a significantly reduced feed-to-gain ratio (F/G) and diarrhea rate of weaned piglets. Fu et al. [48] showed that dietary supplementation with 1.0 × 10^8^ CFU/kg *C. butyricum* significantly improved the intestinal health of weaned piglets, and the feedback on growth performance was a significantly increased average daily gain (ADG) and a significantly reduced diarrhea rate of piglets. One of the possible mechanisms by which dietary *C. butyricum* can promote the growth performance of weaned piglets is that it can promote the secretion of intestinal digestive enzymes (amylase, protease, lipase, and protease), which can degrade macromolecular substances such as carbohydrates, proteins, and lipids in the feed, thereby improving the digestibility of nutrients [27,48,56,74]. Secondly, *C. butyricum* can also improve the intestinal digestion and absorption of nutrients by improving intestinal morphology, and butyric acid produced by *C. butyricum* can be used as a direct energy source for intestinal villi growth, further promoting intestinal villi development and enhancing the intestinal digestion and absorption capacity of nutrients, thus affecting the efficiency of intestinal nutrient digestion and absorption [26,48,59,102].

Although a large number of studies have confirmed the growth-promoting effects of *C. butyricum*, there are also studies showing that *C. butyricum* has no effect on the production performance of weaned piglets [55,74]. This may be related to the different strains of *C. butyricum* used, the different addition amounts, and the different experimental times and environments. Therefore, future research can focus on expanding the screening of *C. butyrate* strains to include better growth-promoting effects and elucidate its growth-promoting effect from the molecular level. For different farming environments, such as poorly ventilated farms, studies can be conducted to reduce the concentration of harmful gases in the air by combining them with other probiotics or functional additives to maintain animal health.

## 7. Conclusions

*C. butyricum* is a kind of green, safe, efficient, and highly resistant probiotic with a variety of biological functions, especially for the regulation of intestinal health. The dietary addition of *C. butyricum* can help maintain the intestinal morphology and microflora homeostasis of piglets, promote intestinal digestion and the absorption of nutrients, enhance the immunity and stress resistance of piglets, and improve the growth performance of piglets. In conclusion, *C. butyricum* exerts a beneficial influence on intestinal health in piglets by regulating the functions of the mechanical barrier, chemical barrier, immune barrier, and microbial barrier.

## Figures and Tables

**Table 1:** Effects of *C. butyricum* on intestinal physical barrier function of piglets.

Weaned Age	Optimal Added Amount	Experimental Period	Significant Effects	References
Intestinal morphology				
21 days	0.4%	35 days	VH↑, and VCR↑	Chen et al. [26]
28 days	5 × 10^5^ CFU/g	14 days	CD↓, and VCR↑	Li et al. [33]
28 days	1.44 × 10^9^ CFU/kg	28 days	VH↑, CD↓, and VCR↑	Wu et al. [37]
28 days	2.5 × 10^9^ CFU/kg	28 days	VH↑	Han et al. [47]
not mentioned	1.0 × 10^8^ CFU/kg	14 days	VH↑, height of microvilli↑	Fu et al. [48]
28 days	6 × 10^9^ CFU/kg	28 days	VCR↑	Wang et al. [50]
28 days	6 × 10^9^ CFU/kg	28 days	VH↑, CD↓, and VCR↑	Wang et al. [51]
21 days	5 × 10^11^ CFU/kg	14 days	VCR↑	Li et al. [58]
23 days	1 × 10^8^ CFU/kg	not mentioned	VH↑, and VCR↑	Zong et al. [59]
Intestinal permeability				
21 days	5 × 10^5^ CFU/g	14 days	DAO↓, D-lactic acid↓	Li et al. [28]
28 days	1.44× 10^9^ CFU/kg	28 days	DAO activity↓	Wu et al. [37]
not mentioned	1.0 × 10^8^ CFU/kg	14 days	DAO↓, D-lactic acid↓	Fu et al. [48]
21 days	500 mg/kg	14 days	endotoxin↓, D-lactic acid↓	Pang et al. [55]
25 days	500 mg/kg	30 days	D-lactic acid↓	Lu et al. [56]
21 days	5 × 10^11^ CFU/kg	14 days	D-lactic acid↓	Li et al. [58]
Tight junctions				
21 days	5 × 10^5^ CFU/g	14 days	ZO-1↑, Claudin-3↑, and Occludin↑	Li et al. [28]
28 days	1.44 × 10^9^ CFU/kg	28 days	*Claudin*-*1*↑, *Claudin*-*2*↑, *Claudin*-*3*↑ and *ZO-1*↑; Claudin3 protein↑	Wu et al. [37]
not mentioned	1.0 × 10^8^ CFU/kg	14 days	ZO-1↑, Claudin-1↑, and Occludin↑	Fu et al. [48]
21 days	500 mg/kg	14 days	*ZO-1*↑, and *Occludin*↑	Pang et al. [55]
25 days	1000 mg/kg	30 days	ZO-1↑	Lu et al. [56]
21 days	5 × 10^11^ CFU/kg	14 days	*ZO-1*↑, and *Occludin*↑	Li et al. [58]
23 days	1 × 10^8^ CFU/kg	not mentioned	*Claudin-1*↑, *Occludin*↑, *ZO-1*↑ and *ZO-2*↑	Zong et al. [59]
28 days	5 × 10^5^ CFU/g	14 days	Claudin-1↑, and *ZO-2*↑	Li et al. [60]

CD: crypt depth; DAO: VCR: villus-height-to-crypt-depth ratio; VH: villus height; ZO-1: zonula occluden-1; ZO-2: zonula occluden-2; “↑” means increase, and “↓” means decrease.

**Table 2:** Effects of *C. butyricum* on intestinal microorganisms of piglets.

Experimental Period	Optimal Added Amount	Significant Result	References
35 days	0.4%	Colon *Bacillus*↑, *Ruminococcaceae UG-003*↑at genus level; colon *Lactobacillus casei*↑, *Parasutterella secunda*↑ at species level	Chen et al. [26]
130 days	1 × 10^12^ CFU/t	*Escherichia coli*↓, *Salmonella*↓, lactic acid bacteria↑	Hu et al. [27]
28 days	1.44 × 10^9^ CFU/kg	Ileal *Antinobacillus*, *Sarcina*, *Clostridium_sensu_stricto_1*, *Terrisporobacter*, *Chloroplast* and *Campylobacter* ↑; colon *Erysipelotrichaceae_UCG_006*↑, *Alloprevotella*, *Intestinibacter* and *Colidextribacter*↓	Wu et al. [37]
28 days	2.5 × 10^9^ CFU/kg	Colon *Streptococcus* and *Bifidobacterium* ↓	Han et al. [47]
14 days	1.0 × 10^8^ CFU/kg	Caecal *Lactobacillus*↑	Fu et al. [48]
28 days	6 × 10^9^ CFU/kg	Colon microbial richness and α diversity ↑	Wang et al. [51]
14 days	5 × 10^5^ CFU/g	Ileal *Escherichia coli* ↓; jejunal and ileal *Lactobacillus* ↑	Li et al. [60]
28 days	0.1%	Faecal *Escherichia coli* count↓, *Lactobacillus* and *Bifidobacterium* count↑	Zhang et al. [73]
21 days	5 × 10^8^ CFU/kg	Faecal Megasphaera, Ruminococcaceae_NK4A214_group and Prevotellaceae_UCG-003↑, Ruminococcaceae_UCG-005↓	Liang et al. [87]
28 days	10 g/kg	Fecal *Selenomonadales* ↑, *Clostridium*↓; lacetic acid-producing bacteria and acetic acid-utilizing bacteria↑	Zhang et al. [98]
28 days	1% *C. butyricum* combined with 5% corn bran	Fecal *Erysipelotrichales*↓; *Clostridiales*↑, *Lactobacillales*↑, *Selenomonadales*↓, *Bacteroidales*↓ at order level	Zhang et al. [99]
21 days	2.0 × 10^8^ CFU/kg body weight	Ileal *Streptococcus* and *Enterococcus*↓	Zhang et al. [100]

“↑” means increase, and “↓” means decrease.

**Table 3:** Growth promoting effect of *C. butyricum* on piglets.

Weaned Age	Optimal Added Amount	Experimental Period	Growth Performance	References
20 ± 2 d	1.25 × 10^11^, 2.50 × 10^11^ or 3.50 × 10^11^ CFU/kg	35 days	ADG, G/F quadratic increased	Casas et al. [20]
21 d	0.4%	35 days	F/G↓, diarrhea score↓	Chen et al. [26]
28 d	1.44 × 10^9^ CFU/kg	28 days	FBW↑, ADG↑, F/G↓, diarrhea rate↓	Wu et al. [37]
28 d	2.5 × 10^8^ or 2.5 × 10^9^ CFU/kg	28 days	F/G↓, average fecal score↓	Han et al. [47]
not mentioned	1.0 × 10^8^ CFU/kg	14 days	ADG↑, diarrhea rate↓	Fu et al. [48]
21 ± 2 d	100 mg/kg	28 days	ADG↑, F/G↓, diarrhea rate↓	Cao et al. [49]
28 d	6 × 10^9^ CFU/kg	28 days	FBW↑, ADG↑, ADFI↑, F/G↓, diarrhea rate↓	Wang et al. [50]
28 d	6 × 10^9^ CFU/kg	28 days	FBW↑, ADG↑, ADFI↑, F/G↓, diarrhea rate↓	Wang et al. [51]
21 d	500 mg/kg	14 days	Diarrhea rate↓	Pang et al. [55]
25 d	250, 500, 1000, 2000 mg/kg	30 days	Diarrhea incidence quadratic decreased	Lu et al. [56]
23 ± 2 d	1.0 × 10^9^ CFU/kg	not mentioned	Diarrhea rate↓	Zong et al. [59]
28 d	5 × 10^5^ CFU/g	14 days	ADG↑, F/G↓	Li et al. [60]
28 d	0.1%	28 days	ADG↑, G/F↑	Zhang et al. [73]
28 d	2.5 × 10^5^ CFU/g	42 days	FBW↑, ADG↑, G/F↑	Takahashi et al. [101]

ADFI: average daily feed intake; ADG: average daily gain; FBW: final body weight; F/G: feed intake-to-gain ratio; G/F: gain-to-feed intake ratio; “↑” means increase, and “↓” means decrease.

## Data Availability

Not applicable.
